# “The angel within the devil”: COVID-19 silver linings

**DOI:** 10.11604/pamj.2021.40.251.32345

**Published:** 2021-12-21

**Authors:** Nuworza Kugbey, Anthony Amoah, Sam-Quarcoo Dotse, Emelia Amoako-Asiedu, Cephas Delalorm, Eric Nyarko-Sampson

**Affiliations:** 1 School of Natural and Environmental Sciences, University of Environment and Sustainable Development, Somanya, Ghana,; 2 School of Sustainable Development, University of Environment and Sustainable Development, Somanya, Ghana,; 3 University of Environment and Sustainable Development, Somanya, Ghana

**Keywords:** COVID-19, healthcare, education, economy, ecosystem

## Abstract

Coronavirus disease (COVID-19) has impacted every aspect of human existence in a variety of ways. However, depending on how we interpret the impact of the pandemic, we may either despair or embrace challenges with hope. Several empirical findings and expert opinions have highlighted the significant negative impact of COVID-19 on economy, health and wellbeing, education, ecosystem and governance around the world. Amid all these negative effects on human existence, we claim that there are some silver linings across several domains such as health and wellbeing, education, eco-system and social connectedness, with the main benefit being adherence to public health measures which will be retained beyond the pandemic.

## Commentary

The COVID-19 has become a threat to human existence with several negative impacts being reported in both developed and developing countries. Countries' economies are under stress with reported deleterious effects on other sectors. In the COVID-19 literature, reports on its negative impacts have been overwhelmingly discussed and analyzed. However, what is unclear, is the evidence of a silver lining amidst the pandemic. This manuscript bridges that gap through a review approach. That is, we discuss the observed impact of COVID-19 with emphasis on the positives in sectors such as healthcare, education and ecosystem with focus on Ghana as an example.

**COVID-19, economy and entrepreneurship**: the outbreak of the Coronavirus (COVID-19) disease is alleged to have started in Wuhan China, an emerging economy, yet its impact on economies has been global hence described as “the greatest” economic shock with severe distortions in the twenty-first century [1]. Most developing and emerging economies have not been spared so far. However, the brighter side of the pandemic on entrepreneurship, savings, innovation and employment among low-income earners cannot be overemphasised. Urban markets in most developing countries especially Africa is said to employ most of the urban population in the cities [2]. In the wake of the pandemic with very critical public health concerns, demand for sanitisers, face mask, detergents, handwashing facilities, water containers and other public health needs rose markedly. As a blessing in disguise, petty traders and other sole sellers as well as small and medium-sized enterprises (SMEs) greatly participated in this line of business. While some low-income earners contributed to the production of personal protective equipment (PPE), some played diverse roles in the value chain to earn decent income. This, together with other factors pushed up the consumer prices which saw a rise in the general price levels [2]. Indeed, all else held constant, the sudden rise in prices inured to the benefit of these producers in the form of higher prices and higher profits. This further attracted many of the unemployed youth into income generating activities within the public health space. To some, this has created a permanent decent job which would not have existed but for the outbreak of the pandemic. Another fact that can hardly be ruled out is increase in private individuals or household´s savings during the COVID-19 pandemic period. This is plausible on grounds that restrictions on social activities, movements and travels are expected to induce reduction in individual or household level spending with a possible reverse of an increase in savings. Against, this background, we argue that economic prospects were evident during the period COVID-19 pandemic.

**COVID-19 and healthcare delivery**: the impact of COVID-19 on healthcare delivery has been documented [3]. Healthcare systems that were not strong experienced severe negative impact of the pandemic. For example, the pandemic has disrupted normal healthcare delivery which already have been bedevilled with issues such as inadequate staffing, lack of equipment, inadequate bed spaces, and poor working conditions. These problems are profound in less developed countries with weak healthcare systems and are only compounded by the pandemic. The number of COVID-19 cases have put pressure on all health systems globally with even advanced economies struggling to cope. However, one of the key lessons from the pandemic which can be counted as positive is the urgent need for investment in healthcare expenditure from all levels of governance. Healthcare delivery has largely moved to accept the use of telemedicine which had received little consideration prior to the emergence of COVID-19. The risk of contracting the virus in addition to already existing medical and other health conditions has resulted in patients and health workers embracing telemedicine which can be harnessed to augment healthcare delivery even after pandemic has disappeared. The good news is that governments and other stakeholders are forced to invest in innovative healthcare delivery strategies such as telemedicine. Medical, psychological and psychosocial support services have now moved to virtual platforms with professionals able to consult and deliver healthcare via these virtual platforms such as zoom, skype among others. Even though there are associated challenges with any innovation, addressing these challenges will strengthen the health systems to withstand future health crises. The pandemic has greatly exposed the lack of attention to the social determinants of health and its subsequent disparities in the impact of the COVID-19 on different populations which will force health expenditure investments to address the inequities in health and healthcare delivery post-COVID. For example, the Ghana Infectious Disease Centre (GIDC) facilitated by the Ghana COVID-19 Private Sector Fund in collaboration with the Ghana Armed Forces at the Ga East Municipal Hospital in Accra and the renovation of the old Shai Osudoku District Hospital in Dodowa are important investments in response to COVID-19 which will continue to help the country in the fight against any future pandemics [4]. In addition, the deployment of artificial intelligence (AI) in healthcare delivery [5] is another positive development we can pick from the pandemic. Finally, the increase in public health measures such as frequent handwashing, cleaning of our surroundings and the use of hand sanitizers [6] also constitute another important benefit of the global pandemic as these measures are adopted worldwide and are likely to become inherent in us even after the pandemic.

**COVID-19 and education**: COVID-19 has resulted in disruption of educational systems leading to urgent adjustment to academic calendars. Students and teachers all over the world are faced with keeping up with academic work in the face of a global challenge which threatens human existence. Evidence suggest that the COVID-19 did not only impact the academic work of students and teachers but also significantly affected their health outcomes [7]. It has also been intimated that the situation is not be different in sub-Saharan Africa especially in developing countries where there are insufficient and ineffective systems to address the challenges associated with the pandemic. Despite the negative consequences of the COVID-19 pandemic on the educational sector, there are several positives which can be harnessed to enhance the educational system. Firstly, the pandemic has led to utilization of innovative pedagogical strategies which were hitherto, limited to a cross-section in the educational sector. Most importantly, the introduction of the online or e-learning systems across the educational systems in both developed and developing countries is a key benefit of the pandemic. Lockdown as a preventive measure has propelled the introduction of information and communications technology (ICT) in the educational sector [8] which has now become an integral part of teaching and learning. Although these online or e-learning have their own challenges in terms of accessibility and internet issues [8], the introduction may force stakeholders to institute measures to address the inequities and weaknesses in the educational system especially in deprived areas with less technological coverage. The use of zoom, blackboard and other learning management systems saves time and eliminate structural barriers in accessing quality education. Consequently, webinars and virtual conferences due to the impact of the pandemic are cost effective and economical especially to researchers in less developed countries who would not have been able to afford physical conferences without funding.

**COVID-19 and ecosystem**: the impact of the COVID-19 on the ecosystem cannot be underestimated as evidence in the extant literature suggest that the pandemic has resulted in adverse environmental consequences such as increased medical waste, haphazard disposal of personal protective equipment compounded by decreased recycling activities [9]. Despite these negative consequences of the pandemic, the preventive measures adopted by most countries such as lockdowns and running shifts at the workplace have had significant impact on environmental pollution. For example, the number of cars on our major roads has reduced during the lockdown periods resulting in low greenhouse gas emissions into the atmosphere. The restrictions on air travels for a period of time are also likely to have saved the ecosystem from the gas emissions leading to improved air quality [9] and may likely impact future climate changes. In addition, major markets, schools, hospitals and places of large gathering have also been fumigated as a control and preventive measure. The fumigation of these places is supposed to be regular exercises but most countries especially less developed ones did not prioritise it until COVID-19 struck. The cleaning of the environment and its consciousness brought about by the pandemic can be seen to be beneficial to mankind. The restrictions on human movement and activities as a result of lockdown have limited indiscriminate disposal of waste that are generated on regular basis. The COVID-19 has also brought to the fore the need for environmental cleanliness which is a major bane in most developing countries. Another key area is the preservation of wildlife with reduced disruption and killing of animals and birds due to restricted human activity [10]. Relatedly, the limited number of tourists visiting important tourist sites is likely to give some respite to the environment and wildlife. This therefore is likely to ensure environmental sustainability as human activities constitute the largest contributor of environmental disruptions.

## Conclusion

COVID-19 has greatly affected several aspects of our lives negatively. However, the era also presented humanity with the opportunity to innovate and improve upon existing facilities to save humanity. The pandemic and its control measures have led to strong social ties or connectedness as more people have recognised the value of collaboration to fight a common enemy. We need to take advantage of opportunities presented in the healthcare, education, and other sectors to develop resilience to face any unforeseen future disasters.
